# A Highly CO_2_-Sensitive Wood-Based Smart Tag for Strawberry Freshness Monitoring

**DOI:** 10.3390/polym16202900

**Published:** 2024-10-15

**Authors:** Jin Xu, Yuping Ning, Yalu Yun, Xiling Cheng, Jian Li, Lijuan Wang

**Affiliations:** Key Laboratory of Bio-Based Materials Science and Technology of Ministry of Education, Northeast Forestry University, No. 26 Hexing Road, Xiangfang District, Harbin 150040, China; nefujinx@163.com (J.X.); 18646211961@163.com (Y.N.); 15776658571@163.com (Y.Y.); 17603448533@163.com (X.C.); nefulijian@163.com (J.L.)

**Keywords:** poplar-based smart tag, delignification, CO_2_ indicator, pH sensitivity, strawberry freshness

## Abstract

Smart tags are used for monitoring the freshness of foods. However, they often lack significant color changes, and their accuracy needs to be improved. In this study, a poplar veneer with a natural pore structure was selected as a matrix to prepare a smart tag with high pH sensitivity for tracking the freshness of strawberries. The delignified veneer was modified using 2,3-epoxypropyltrimethylammonium chloride (EPTAC) to be given positive charges to adsorb bromothymol blue (BTB) through electrostatic interactions. The adsorption capacity of the veneer reached 7.0 mg/g at 50 °C for 4 h, and the veneer showed an obvious blue color. The smart tags exhibited distinct color changes at different pHs and showed quick color changes in response to acetic acid. As the freshness of strawberries decreased, the color of the smart tags changed from blue to yellow-green, which indicated that the accuracy was high. In this study, an effective method was fabricated to prepare a highly sensitive tag, promoting popular application to ensure food quality.

## 1. Introduction

Fruits, rich in vitamins, organic acids, dietary fiber, phenolic compounds, minerals, and other nutrients, are very beneficial to human health and are an indispensable part of human diet [1]. However, fruits with high water content and delicate tissues are highly susceptible to deterioration which will lead to a reduction in nutritional value [2] and may even cause potential risks to human health. Therefore, there is an urgent need for methods to assess the quality of fruits to ensure their nutritional value and safety. The traditional testing of the freshness of fruits is mainly based on microbiological, physical, and chemical analyses and sensory assessments [3,4]. These methods are usually time-consuming, labor-intensive, and susceptible to subjective factors, which limits their popular application [5,6]. In recent years, smart tags have been widely studied due to timely, accurate, and intuitive monitoring. Among smart tags, CO_2_-sensitive tags have received more and more attention due to their ability to produce visible color changes as sensing the quality of fruits [7,8].

Smart tags generally consist of a matrix of natural biopolymers and sensitive dyes. Natural dyes from plant tissues, such as anthocyanins, betalains, curcumin, and alizarin, have shown potential for monitoring freshness in meats like pork [9], chicken [10], and fish [11,12]. Although natural dyes are healthy and non-toxic, their stability and reliability can be compromised [8]. For example, blueberry extract (anthocyanin) as a sensitive dye was added into a corn starch/nanoclay film prepared by extrusion and thermoforming techniques [13]. However, the film did not show pH sensitivity probably because the shear force and high pressure and temperature in the extruder led to the decomposition of the blueberry extract. Compared to natural dyes, synthetic dyes such as bromothymol blue, methyl red, and bromocresol green are more stable and can produce significant color changes at different pHs [14,15]. They have been used to monitor fruit freshness. For instance, bromothymol blue was fixed in a mixed matrix of sodium carboxymethyl cellulose and carrageenan to reflect the freshness of fresh-cut papaya [16].

Natural biopolymer matrices such as polysaccharides and proteins have been used to fix dyes to obtain indicator films using the casting method [17,18,19]. Succinylated chitosan and hydroxypropyl chitosan were mixed with a mixture of bromothymol blue and methyl red dyes, dried and molded in a thermostat, and then used to indicate the freshness of fruits [20]. The uniformity of the mixed indicator in the chitosan-based composite film decreased when the indicator concentration was greater than 1 wt%. Moreover, indicator films are generally prepared using the casting method, which suffers from the problems of the easy introduction of impurities, unfavorable bubbles, and the evaporation of solvents, which affect the dispersion of dyes in the drying process. In addition, they are usually highly transparent, and the colors are easily disturbed by the background [21].

To resolve these problems, wood as a promising matrix has been explored in our previous study because it is colorless, opaque, sensitive to humidity, mechanically strong, and stable in anchoring dyes. Poplar veneer was bleached and carboxylated by using alkaline H_2_O_2_ to adsorb mulberry anthocyanins to prepare a smart tag [22]. Mi et al. quaternized poplar veneer by a two-step process for the adsorption of bromothymol blue (BTB) to prepare a smart tag for monitoring the freshness of milk [23]. However, the retention of most of the lignin made the response slow, and the two-step process was complex. Consequently, it is necessary to develop a smart tag with fast response via a simpler preparation process.

Wood has a certain degree of permeability due to its naturally porous structure, which is conducive to the diffusion of gases and liquids inside [24]. Therefore, increasing the porosity of poplar veneer is an effective way to improve the sensitivity of tags. Based on the compositional structure of wood, the selective removal of lignin can increase its porosity [25]. So, smart tags can sense the pH changes in the surrounding environment more quickly. In addition, a suitable quaternary ammonium salt was directly selected for the cationization of the wood veneer, avoiding the preparation process of the quaternized reaction solution and the generation of toxic substances.

**Figure 1:**
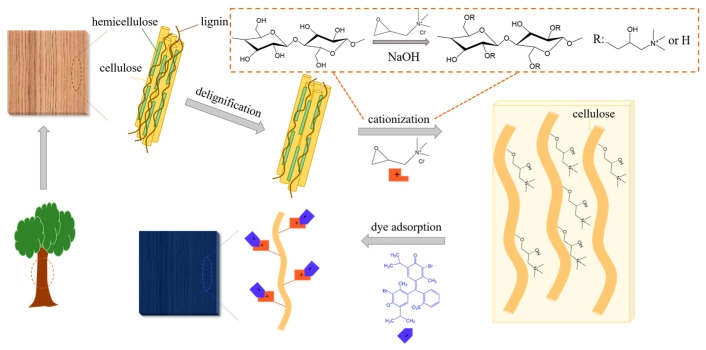
A mechanistic diagram of the preparation process and microstructure of the smart tag.

In this study, we used a poplar veneer as a matrix to adsorb an anionic dye (bromothymol blue) to prepare a CO_2_-sensitive smart tag for monitoring the freshness of fruits. To this end, the poplar veneer was delignified firstly [26] and then cationized by 2,3-epoxypropyltrimethylammonium chloride (EPTAC) to obtain a positively charged wood veneer to adsorb anionic dyes through electrostatic action (Figure 1) [27,28,29]. BTB is an acid–base indicator that can be protonated by H^+^ dissociated from carbonic acid produced from CO_2_ in the presence of water molecules, resulting in a color change. To ensure the viability of smart tags, pH and acid gas responses were tested, and the application for monitoring strawberry quality was investigated.

## 2. Materials and Methods

### 2.1. Materials

Poplar veneers (30 mm × 30 mm × 0.85 mm) were obtained from Dehua TB New Decoration Material Co., Ltd. (Huzhou, China). Sodium chlorite (NaClO_2_, AR) was obtained from Tianjin Damao Chemical Reagent Factory (Tianjin, China). Glacial acetic acid and sodium hydroxide (NaOH, AR) were provided by Tianjin Dongli District Tianda Chemical Reagent Factory (Tianjin, China). 2,3-epoxypropyltrimethylammonium chloride (EPTAC, ≥95%) was acquired from Shandong Keyuan Biochemical Co., Ltd. (Shandong, China). Bromothymol blue (BTB, AR) was obtained from Tianjin Guangfu Fine Chemical Research Institute (Tianjin, China). Fresh strawberries were purchased from a local supermarket (Harbin, China).

### 2.2. Preparation of Smart Tags

#### 2.2.1. Delignification of Wood

Poplar veneers (PVs) were immersed in 2 wt% NaClO_2_ aqueous solution, and the pH was adjusted to about 4.6 with glacial acetic acid, and then the veneers were treated in boiled solution for 40 min to remove lignin. The delignified wood veneers (DWVs) were washed with deionized water and freeze-dried.

#### 2.2.2. Cationic Wood Veneer

The delignified wood veneer was placed in 2 wt% NaOH aqueous solution at room temperature for 1.5 h, followed by the addition of EPTAC to achieve a concentration of 0.5 mol/L. Then, the reaction was carried out at 65 °C for 3 h to obtain a cationic wood veneer (CWV), which was washed with deionized water and freeze-dried.

#### 2.2.3. Preparation of BTB Fixed Smart Tag (BT)

Bromothymol blue was dissolved in 0.05 mol/L NaOH aqueous solution to obtain the dye solution. The cationic wood veneer was immersed in BTB/NaOH (1 g/L) solution at 30 °C and 50 °C for 1 h, 2 h, and 4 h, respectively. Afterward, surface coloration was removed with 0.05 mol/L NaOH, followed by extensively washing with deionized water and freeze-drying to prepare smart tags.

A UV-Vis spectrophotometer (UV-2600, Shimadzu, Kyoto, Japan) was used to obtain the standard curve of BTB in 0.05 mol/L NaOH aqueous solution, the absorbance of BTB solution before and after adsorption was determined at 615.8 nm, and the concentration was calculated; the amount of BTB adsorbed (Q) was obtained according to Equation (1):

(1) $$Q=\frac{\left(C_{0\;}-{\;C}_{e}\right)}{m}\;\times \;V$$

where Q (mg/g) represents the adsorption capacity of BTB; C_0_ and C_e_ (mg/mL) are the concentration of BTB solution before and after adsorption, respectively; m (g) is the weight of CWV; and V (mL) represents the volume of the BTB/NaOH solution used.

### 2.3. Characterizations of Structure and Morphology

Morphological characteristics of poplar veneer, delignified wood veneer, cationic wood veneer, and smart tags in a cross-section were observed at 500, 1000, and 5000 magnifications using a scanning electron microscope (SEM) (Thermo Fisher, Waltham, MA, USA). A cross-section of the samples was cut using a slicer (Leica SM2010R, Leica, Wetzlar, Germany), and the sliced samples were fixed on sample stages with conductive adhesive and sprayed with gold before observation. Functional groups were determined in the ATR model using a Fourier transform infrared spectrometer (FTIR) (Nicolet 6700, Thermo Fisher, Waltham, MA, USA) in the range of 4000–500 cm^−1^. Elements were evaluated using an X-ray photoelectron spectrometer (XPS) (Thermo Fisher, Waltham, MA, USA). The crystalline structure of wood veneer was studied using an X-ray diffractometer (XRD) (Shimadzu, Kyoto, Japan).

### 2.4. Porosity and Pore Growth Rate

Poplar veneers were initially dried to a constant weight; then, the length, width, thickness, and weight of the veneers were measured. Delignification was performed using the method in Section 2.2.1, and the veneers were boiled for 10 min, 20 min, 30 min, and 40 min, respectively, to obtain DWVs. Their length, width, thickness, and weight were measured separately. The porosity and pore growth rate of DWV were calculated according to Equations (2) and (3):

(2) $$P=\left(1\;-\frac{\rho }{\rho_{0}}\right)\times \;100\%$$

(3) $$P_{i}=\left(\frac{P_{e}-P_{0}}{P_{0}}\right)\times \;100\%\;$$

where P (%) represents the porosity of the veneer, ρ and ρ_0_ represent the dried density and substantial density of the poplar [30], P_i_ (%) represents the pore growth rate of the veneer after delignification, and P_e_ (%) and P_0_ (%) represent the porosity of DWV and PV, separately.

### 2.5. Moisture Absorption Test

The smart tags were cut to the size of 7.5 mm × 7.5 mm and placed in a desiccator with a relative humidity of 11%, 43%, and 75% for 24 h, and the weight changes were recorded. The moisture absorption rate (M%) was calculated from Equation (4):

(4) $$M\%=\frac{m_{e\;}-{\;m}_{0}}{m_{0}}\;\times \;100\%$$

where m_0_ represents the initial weight of the sample, and m_e_ represents the weight of the sample after 24 h of moisture absorption.

### 2.6. pH Response Test

The smart tags (7.5 mm × 7.5 mm) were immersed in buffer solutions of different pHs (pH = 2.0, 4.0, 6.0, 7.0, 8.0, 9.0) until the color of the tags did not change. Then, a hand-held colorimeter was used to determine the color parameters (L*, a*, b*), and ΔE was calculated according to Equation (5):

(5) $$\Delta E=\sqrt{{\left(L^{*}-{{\;L}_{0}}^{*}\right)}^{2}+{\left(a^{*}-{{\;a}_{0}}^{*}\right)}^{2}+{\left(b^{*}-{{\;b}_{0}}^{*}\right)}^{2}}$$

where L_0_*, a_0_*, and b_0_* are the original colorimetric parameters of the tag.

### 2.7. Acid Gas Response

The smart tags (7.5 mm × 7.5 mm) containing constant humidity (11%, 43%, and 75%) were attached to the top of a sealed plastic container (450 mL), and 0.1 mL of acetic acid was injected into the container using a syringe. Subsequently, the color changes in the tags were recorded with the camera of a mobile phone at specified intervals (30 s, 60 s, 300 s, 600 s, and 900 s).

### 2.8. Application of Smart Tags to Track Strawberry Freshness

Fresh strawberries (approximately 305 g) purchased from a local supermarket were placed in an airtight container and stored at room temperature with a smart tag attached to the top of the container. As the color of the tag changed, a CO_2_ headspace tester was used to measure the CO_2_ content in the container, and a GY-4 hand-held fruit hardness tester with an 8 mm diameter probe was applied to measure the hardness of strawberries. The total acid content of strawberries was determined by acid–base indicator titration according to Chinese GB 12456-2021.

## 3. Results and Discussion

### 3.1. Characterization of PV, DWV, CWV, and Smart Tag

**Figure 2:**
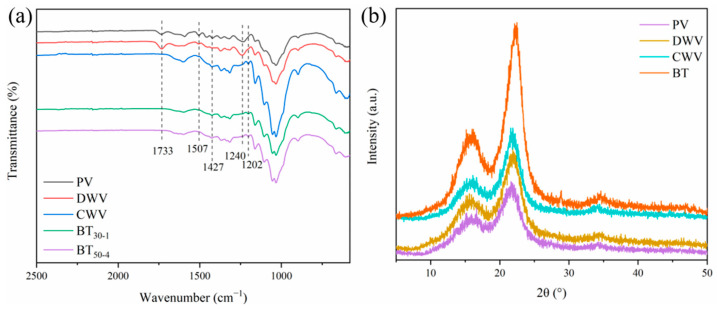
FTIR spectra (portion) (**a**) and XRD patterns (**b**) of PV, DWV, CWV, and smart tag.

The FTIR spectra of PV, DWV, CWV, and the smart tag are shown in Figure 2a and Figure S1. The characteristic peak of hemicellulose at 1733 cm^−1^ (C=O stretching vibration in hemicellulose) increased after delignification and gradually disappeared after quaternization, probably due to the relative elevation of hemicellulose after lignin was removed, and then hemicellulose was removed again during quaternization due to the action of NaOH. The peaks at 1507 cm^−1^ and 1240 cm^−1^ corresponded to the aromatic ring backbone stretching vibration of lignin and the C-O vibration of acyl groups in lignin and hemicellulose, respectively [31,32]. They diminished or disappeared after delignification and quaternization, indicating the removal of lignin and hemicellulose. After quaternization, a new peak at 1427 cm^−1^ appeared, which might be due to the C-N stretching vibration of the accessed quaternary ammonium group [33]. The presence of another new peak at 1202 cm^−1^ may be due to C-O-C vibrations in EPTAC remaining in the wood after quaternization.

Figure 2b shows the XRD patterns of PV, DWV, CWV, and the smart tag. The characteristic peaks at 15.68°, 21.90°, and 34.32° in Figure 2b corresponded to the diffraction of (101), (002), and (040) crystal planes in cellulose I, respectively [34]. After delignification and quaternization, DWV and CWV still showed the three classical diffraction peaks of cellulose I and exhibited the same crystalline structure as the poplar veneer, indicating that delignification and quaternization had little effect on the crystal structure of cellulose. After adsorbing BTB, the crystal structure of the smart tag was similar to that of CWV because the dye bound to the veneer by electrostatic action without affecting the crystal structure of cellulose.

**Figure 3:**
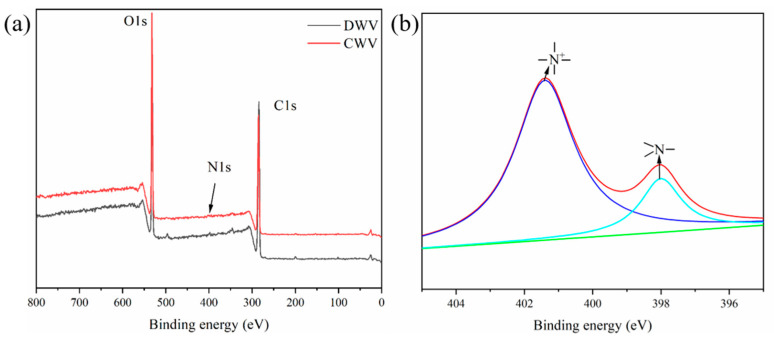
Full spectra of XPS for DWV and CWV (**a**); high-resolution XPS spectrum of N1s in CWV (**b**).

The full spectra of the XPS of DWV and CWV are shown in Figure 3a, and the N1s spectrum of CWV is presented in Figure 3b. The XPS spectrum of N1s was curve-fitted to two peaks located at 397.98 eV and 401.38 eV, which corresponded to N1s in the nitrogen compounds originally present in poplar and N^+^ in EPTAC, respectively, proving that EPTAC was successfully introduced into the wood.

**Figure 4:**
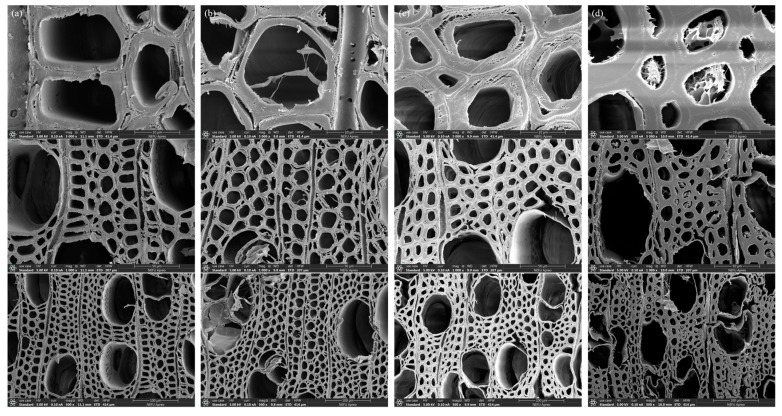
SEM images of PV (**a**), DWV (**b**), CWV (**c**), and the smart tag (**d**).

Figure 4 shows the microscopic morphology of PV, DWV, CWV, and the smart tag, in which a similar porous structure can be seen. The poplar veneer had a rough surface, dense intercellular layer, and firm conduit structure (Figure 4a). After delignification (Figure 4b), the cracks in the intercellular layer of DWV became larger, and the pores increased. Following quaternization (Figure 4c), cracks appeared on wood fibers. Moreover, granular structures were observed on the fibers, which might be the aggregates of quaternary ammonium salts. After BTB staining (Figure 4d), the conduit collapsed, the cracks on the wood fibers disappeared, and the fibers became thickened, which might have been caused by BTB being adsorbed in them. And agglomerates appeared on the wood fibers, which might be caused by the shedding and gathering of the surface layer of wood fibers.

### 3.2. Porosity

**Figure 5:**
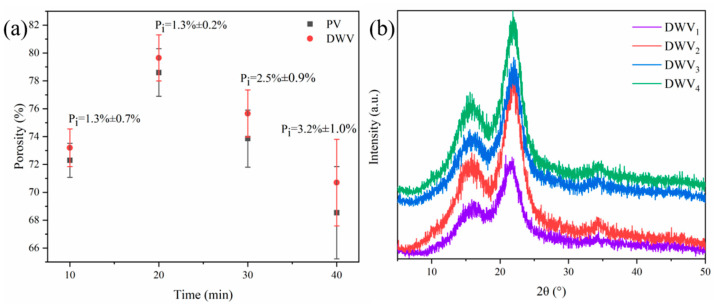
Porosity of PV and DWV, and pore growth rate (P_i_) of DWV (**a**); XRD of wood veneers with different delignification times (**b**).

After delignification, DWV has a whiter color and a rougher surface, and the porosity, specific surface area, and hydrophilicity of the veneer were improved, which was conducive to its moisture absorption and permeability. Figure 5a shows that as the time of delignification increased, the porosity of delignified veneer gradually became larger compared with that of the poplar veneer. After 10 min of delignification, the pore growth rate of the veneer was 1.3%, and when the time was extended to 40 min, the pore growth rate increased to 3.2%. When the delignification time was further extended, the mechanical strength of the wood veneer in the subsequent process was severely reduced and even fell apart, so 40 min was used as the optimal one. DWV had a larger porosity, and a more open porous structure made the wood much more hygroscopic and permeable and more sensitive to gases during application. In addition, delignification exposed more hydroxyl reactive functional groups on the surface of the veneer, which facilitated the grafting reaction of EPTAC with hydroxyl groups. The crystalline structure of DWV_1–4_ is similar to that of the original poplar veneer (Figure 5b), demonstrating that different delignification times did not disrupt the ordered arrangement of cellulose molecules.

### 3.3. Dye Adsorption

**Figure 6:**
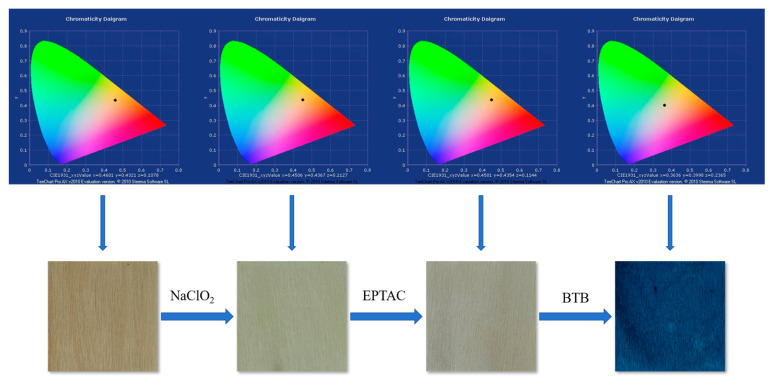
Color optical fiber spectra and photographs of PV, DWV, CWV, and the smart tag (BT_50-4_).

The smart tags were prepared by combining CWV with negatively charged BTB by electrostatic interactions. Different times (1 h, 2 h, and 4 h) and temperatures (30 °C, 50 °C) were set to explore their effects on the adsorption capacity of BTB on CWV (Table 1). The data indicated that the adsorption capacity of CWV for BTB increased gradually with the increase in time and temperature. At 50 °C, the adsorption capacity reached 7.0 mg/g after 4 h, and the smart tag obviously changed to blue (Figure 6). The results demonstrated that the adsorption capacity of CWVs was positively correlated with time and temperature, and the effect of time was slightly obvious. So, the adsorption conditions can be adjusted on demand. Additionally, the FTIR spectra of smart tags with different adsorption conditions were similar to those of the cationic wood veneer (Figure S2), suggesting that BTBs bound to the cationic wood veneers via electrostatic interactions had no effect on the skeletal structure of the poplar veneer. Subsequently, BT_30-1_ and BT_50-4_ (the adsorption conditions of the tags were 30 °C, 1 h and 50 °C, 4 h, respectively) were used to explore the response of the tags and monitor strawberry freshness to ensure that the adsorption capacity of 3.4–7.0 mg/g did not have a large effect on the trend in the tag response.

### 3.4. Moisture Absorption

**Figure 7:**
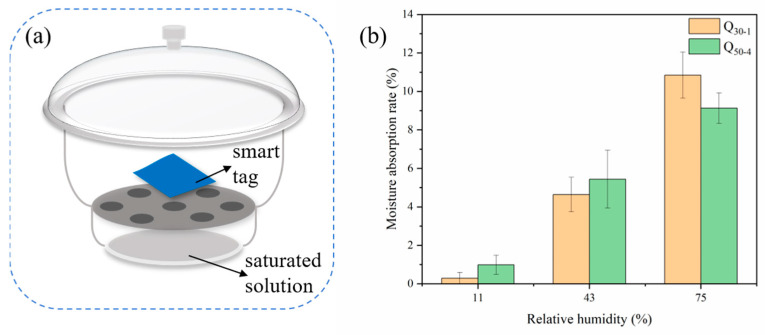
Dryer device (**a**); moisture absorption rate of smart tags at 11%, 43%, and 75% relative humidity (**b**).

When the relative humidity increased from 11% to 75%, the moisture absorption rate of BT_30-1_ and BT_50-4_ rose from 0.3% to 10.9% and from 1.0% to 9.1% (Figure 7), respectively, indicating that the adsorption of dyes had little effect on the moisture adsorption capacity of the tags. For CO_2_-sensitive smart tags, low humidity was not conducive to color changes because fewer water molecules collected on the surface of the wood would result in less H^+^ produced by the dissociation of carbonic acid formed by the combination of CO_2_, so smart tags should be used in high-humidity conditions.

### 3.5. pH Response

**Figure 8:**
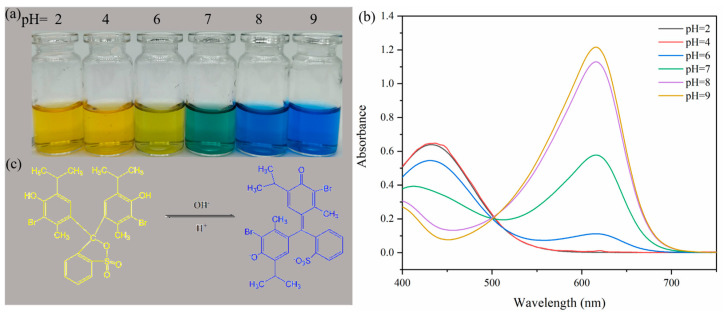
Color changes (**a**) and UV spectra (**b**) of BTB under different pH buffer solutions; structural changes in bromothymol blue in alkaline and acidic solutions (**c**).

BTB is an acid–base indicator that exhibits different colors in different pH environments: yellow in acidic conditions (pH < 6.0) and blue in alkaline conditions (pH > 7.6) (Figure 8a,c) [35,36].

Figure 8 shows the color changes (Figure 8a) and UV–visible spectra (Figure 8b) of BTB in different pH buffer solutions. At pHs 2 and 4, BTB was yellow in buffer solutions. With the increase in pH, the color of BTB shifted from yellow to blue, showing yellow-green at pH 6, green at pH 7, and blue at pHs 8 and 9. Additionally, the maximum UV–visible absorption peak of BTB gradually shifted from 432 to 615 nm. Table 2 shows the color and parameter changes in the smart tags with two adsorption amounts at different pH values. The smart tags were yellow in an acidic environment, and as the pH became higher, the color of the tags started to turn green and finally blue in an alkaline environment. Concurrently, the tag color parameter b* gradually decreased to a negative value, indicating a gradual increase in the blue tone, and when ΔE > 5, a color change could be clearly seen.

### 3.6. Acetic Acid Response

**Figure 9:**
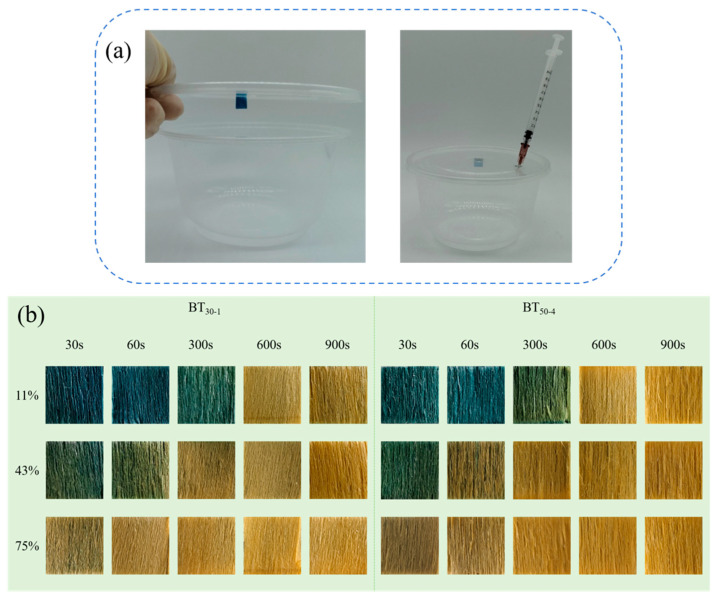
Acetic acid response device (**a**); color changes in smart tags in acetic acid environment with relative humidity of 11%, 43%, and 75% (**b**).

A syringe was used to inject 0.1 mL of acetic acid into a closed container labeled with a smart tag to simulate the response to acidic gas [37]. As shown in Figure 9, with the extension of response time, the tag gradually changed from blue to yellow. As the relative humidity increased, the time required for the tags to change color decreased. At 11% humidity, the tag changed to yellow in approximately 900 s, whereas at 75% humidity, it changed to yellow in 300 s. This was because more water molecules collected on the surface of the tag, which accelerated the hydrolysis of acetic acid to produce H^+^, resulting in a faster color change [15]. The results revealed that the smart tag had a more sensitive response.

### 3.7. Application of Smart Tags in Strawberry Preservation

During the storage of strawberries, their respiration and the microbial reproduction on the surface will produce CO_2_, and an increase in CO_2_ content indicates that the freshness of strawberries decreases [38]. CO_2_ is an acidic gas that combines with moisture in the headspace of the package to form carbonic acid. H^+^ produced by the dissociation of the carbonic acid protonates BTB molecules, the pH in the space decreases, and the smart tags have a color change, thus reflecting the change in strawberry freshness [7].

**Figure 10:**
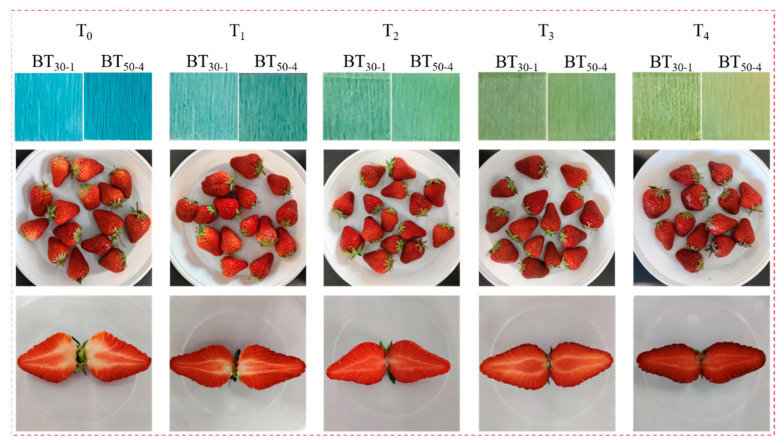
A schematic diagram of the color of the smart tag and the status of the strawberry during storage (T_0_–T_4_ stand for storage time (h)).

Figure 10 shows the color change in the smart tags during the strawberry storage. Initially (T_0_), the strawberries were fresh, and the tags were blue. At this moment, the hardness, CO_2_ content, and total acid content of strawberries were 2.51 kg/cm^2^, 0.65%, and 7.870%, respectively (Table 3). The metabolic activities of strawberries consume organic acids to release CO_2_, and these changes can reflect the freshness of strawberries. With the prolongation of storage time (T_1_–T_3_), the hardness and total acid content of strawberries declined while the CO_2_ concentration in the container increased, indicating that the freshness of the strawberries was decreasing, and the color of the tags changed accordingly (light blue—cyan—light yellow-green). At T_3_, strawberries lost water and softened. At T_4_, the tags changed to yellow-green, the hardness and total acid content of strawberries decreased to 1.64 kg/cm^2^ and 6.465%, respectively, and the CO_2_ concentration rose to 97.48%. The strawberries showed visible rot; they were no longer fresh at this moment. Therefore, the smart tag was timely and effective for monitoring the freshness of strawberries. Compared to other smart tags tracking the freshness of fruits, such as the CO_2_-sensitive antimicrobial bilayer films prepared by Li et al. [7], the smart tags in this study exhibited a more obvious color change. The tags have potential to be applied to other fruits because the respiration of most fruits and microbial reproduction are accompanied by the production of CO_2_.

## 4. Conclusions

A highly CO_2_-sensitive tag for monitoring strawberry freshness was prepared by grafting quaternary ammonium salt groups to give the delignified poplar veneer positive charges to adsorb BTB via electrostatic interactions. The FTIR and XPS results confirmed the successful grafting of the quaternary ammonium groups. After staining, the poplar veneer exhibited an obvious blue color, indicating that BTB was successfully anchored in the veneer through electrostatic interactions. The smart tag exhibited high pH sensitivity, could produce obvious color changes in different pHs, and had a positive response to acetic acid. In addition, when the smart tag was applied to monitor strawberry freshness, as the CO_2_ concentration in the container increased, the smart tag exhibited a significant color change from the initial blue color to yellow-green, which could warn consumers that the strawberries were not fresh. BT_30-1_ and BT_50-4_ did not have a significant effect on the monitoring of strawberry freshness; thus, BT_30-1_ was a better choice due to its efficient preparation. In this study, a highly sensitive smart tag was developed to provide a new path for monitoring the freshness of food and a new strategy for the processing and utilization of low-quality wood.

## Figures and Tables

**Table 1:** The adsorption capacity of the veneers for BTB under different times and temperatures.

Time (h)	Temperature (°C)	Q (mg/g)
1	30	3.4 ± 0.7 ^c^
	50	3.7 ± 0.6 ^c^
2	30	4.6 ± 0.5 ^bc^
	50	5.2 ± 1.3 ^b^
4	30	5.4 ± 0.4 ^b^
	50	7.0 ± 1.0 ^a^

Data are mean ± standard deviation, and means with different letters within same column are significantly different (*p* < 0.05).

**Table 2:** Color changes and color parameters of smart tags at different pH values.

BT	pH	Photo	L*	a*	b*	ΔE
BT_30-1_	2	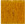	77.5 ± 0.8 ^a^	13.0 ± 0.3 ^a^	53.1 ± 1.4 ^a^	43.3 ± 0.4 ^a^
	4	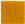	78.2 ± 0.4 ^a^	11.7 ± 0.8 ^b^	48.9 ± 1.7 ^b^	41.6 ± 0.4 ^b^
	6	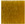	73.3 ± 0.6 ^b^	3.9 ± 0.2 ^c^	44.0 ± 0.6 ^c^	35.0 ± 0.8 ^c^
	7	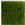	65.8 ± 2.1 ^c^	−8.2 ± 0.5 ^d^	34.3 ± 1.0 ^d^	27.1 ± 0.7 ^d^
	8	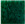	54.2 ± 1.4 ^d^	−19.0 ± 0.5 ^f^	4.2 ± 1.0 ^e^	21.9 ± 0.6 ^e^
	9	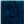	48.8 ± 1.1 ^e^	−16.4 ± 0.2 ^e^	−20.2 ± 0.2 ^f^	20.3 ± 0.2 ^f^
BT_50-4_	2	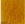	78.0 ± 0.2 ^a^	14.5 ± 0.2 ^a^	53.8 ± 1.3 ^a^	43.6 ± 0.4 ^a^
	4	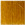	76.7 ± 0.9 ^b^	13.6 ± 0.5 ^b^	50.4 ± 1.5 ^b^	41.8 ± 0.4 ^b^
	6	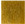	72.5 ± 0.8 ^c^	3.7 ± 0.1 ^c^	42.0 ± 1.0 ^c^	34.4 ± 0.6 ^c^
	7	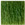	64.8 ± 1.3 ^d^	−9.2 ± 0.4 ^d^	28.6 ± 0.6 ^d^	26.4 ± 0.4 ^d^
	8	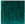	54.3 ± 1.9 ^e^	−18.1 ± 0.8 ^f^	1.5 ± 2.6 ^e^	24.0 ± 0.2 ^e^
	9	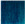	47.5 ± 0.6 ^f^	−13.3 ± 0.7 ^e^	−22.3 ± 0.7 ^f^	22.2 ± 0.5 ^f^

Data are mean ± standard deviation, and means with different letters within same column are significantly different (*p* < 0.05).

**Table 3:** Changes in strawberry hardness, CO_2_ concentration, and total acid content with storage time.

Storage Time (h)	Hardness (kg/cm^2^)	Concentration of CO_2_ (%)	Content of Total Acids (%)
T_0_ (0)	2.51 ± 0.32 ^a^	0.65 ± 0.07 ^e^	7.870 ± 0.00 ^a^
T_1_ (7)	1.95 ± 0.29 ^b^	26.56 ± 0.38 ^d^	7.027 ± 0.40 ^b^
T_2_ (23)	1.92 ± 0.21 ^b^	47.05 ± 0.09 ^c^	6.746 ± 0.00 ^bc^
T_3_ (48)	1.64 ± 0.26 ^b^	87.63 ± 0.63 ^b^	6.184 ± 0.00 ^d^
T_4_ (95)	1.64 ± 0.32 ^b^	97.48 ± 0.01 ^a^	6.465 ± 0.00 ^cd^

Data are mean ± standard deviation, and means with different letters within same column are significantly different (*p* < 0.05).

## Data Availability

The original contributions presented in the study are included in this article/Supplementary Materials; further inquiries can be directed to the corresponding author.

## Supplementary Materials

The following supporting information can be downloaded at: https://www.mdpi.com/article/10.3390/polym16202900/s1, Figure S1: FTIR spectra of PV, DWV, CWV, and the smart tag; Figure S2: FTIR spectra of the smart tags obtained under different adsorption times and temperatures.
