# Supplementary material for: A Highly CO2-Sensitive Wood-Based Smart Tag for Strawberry Freshness Monitoring
Source: Polymers (Basel). 2024 Oct 15;16(20):2900. doi: 10.3390/polym16202900 (PMC11511562; doi:10.3390/polym16202900)
Supplement: Supplementary file 1 [file polymers-16-02900-s001.zip › polymers-3242123-supplementary.pdf]

Supporting information

for

A highly CO_2-sensitive wood-based smart tag for strawberry freshness monitoring

Jin Xu, Yuping Ning, Yalu Yun, Xiling Cheng, Jian Li and Lijuan Wang *

Key Laboratory of Bio-based Materials Science and Technology of Ministry of Education, Northeast Forestry University, No. 26 Hexing Road, Xiangfang District, Harbin 150040, P. R. China; nefujinx@163.com (J.X.); 18646211961@163.com (Y.N.); 15776658571@163.com (Y.Y.); 17603448533@163.com (X.C.); nefulijian@163.com (J.L.); donglinwlj@163.com (L.W.)

* Correspondence: donglinwlj@163.com; Tel.: 86-451-82191693

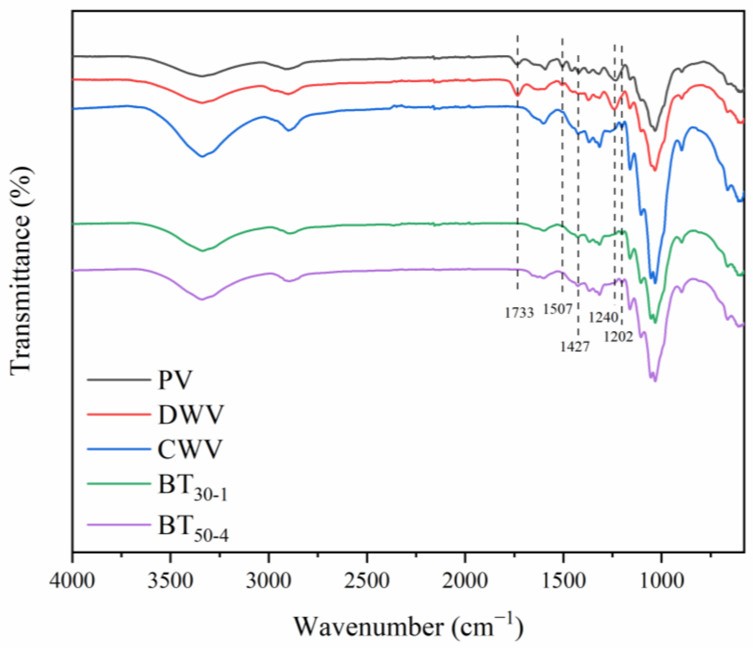

Figure S1. FTIR spectra of PV, DWV, CWV, and the smart tag.

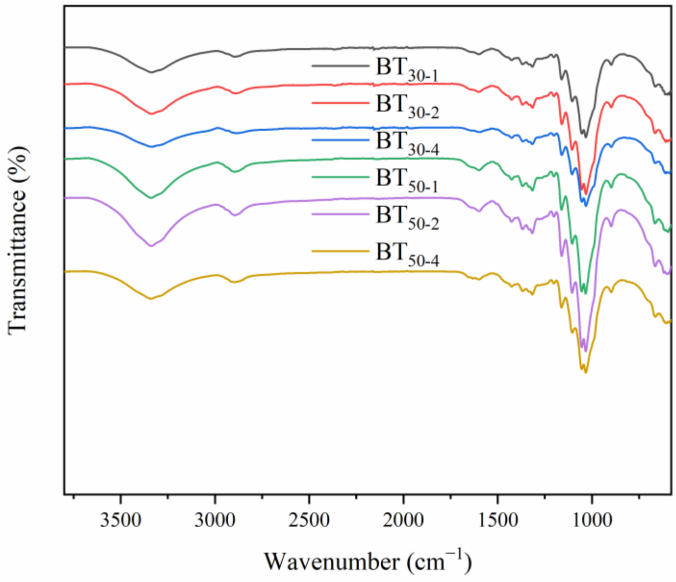

Figure S2. FTIR spectra of the smart tags obtained under different adsorption times and temperatures.
